# Community Detection in Signed Networks: the Role of Negative ties in Different Scales

**DOI:** 10.1038/srep14339

**Published:** 2015-09-23

**Authors:** Pouya Esmailian, Mahdi Jalili

**Affiliations:** 1 Department of Computer Engineering, Sharif University of Technology, Tehran, Iran; 2 School of Electrical and Computer Engineering, RMIT Universiy, Melbourne, Australia

## Abstract

Extracting community structure of complex network systems has many applications from engineering to biology and social sciences. There exist many algorithms to discover community structure of networks. However, it has been significantly under-explored for networks with positive and negative links as compared to unsigned ones. Trying to fill this gap, we measured the quality of partitions by introducing a Map Equation for signed networks. It is based on the assumption that negative relations weaken positive flow from a node towards a community, and thus, external (internal) negative ties increase the probability of staying inside (escaping from) a community. We further extended the Constant Potts Model, providing a map spectrum for signed networks. Accordingly, a partition is selected through balancing between abridgment and expatiation of a signed network. Most importantly, multi-scale spectrum of signed networks revealed how informative are negative ties in different scales, and quantified the topological placement of negative ties between dense positive ones. Moreover, an inconsistency was found in the signed Modularity: as the number of negative ties increases, the density of positive ties is neglected more. These results shed lights on the community structure of signed networks.

During the last decade, there has been an ever-growing interest in community structure of real-world networks^1^,^2^. A community structure is observed on a network when relations are sparse, and there exists a mechanism driving density heterogeneity^3^,^4^. Indeed, grouping a network into modules of relatively high density provides novel insights into characteristics of a network^5^,^6^,^7^,^8^,^9^. However, the mainstream of this trend is mostly involved with only positive relations, where the links are absorptive, and thus “more links” means “more closeness”. In parallel with this trend, there has been some attempts towards community detection in networks with both positive and negative relations^1^,^10^,^11^. As a result, the objective is to partition a network into modules of relatively high density with as few internal negative ties as possible. Hence, this is different from those that are not bound to the density constraint, e.g., Correlation Clustering^12^.

A community is a group of tightly knitted nodes that are weakly connected to the rest of the network. This is not a *definition* per se, nonetheless it is the one upon which literature is mostly agreed, and can be further refined into specific definitions^13^. For signed graphs, the extension is straightforward by adding “as few internal negative ties as possible” criterion. Despite a vast literature on community detection, there has been few attempts towards signed networks^1^. Nonetheless, as the main step, Traag and Bruggeman generalized the modularity-alike objectives to signed graphs, as they have straightforward and intuitive signed counterparts^14^. However, the generalized form introduces a set of crucial parameters that cannot be trivially set. For the case of modularity, the objective function proposed by Gómez *et al*.^15^ is as:


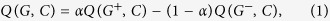


where *G*^+^ (*G*^−^) is the positive (negative) subgraph of *G*, *C* is the partition, and 0 ≤ *α* ≤ 1 is the relative importance of positive subgraph compared to the negative one, which has been set to 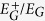
15. This objective function rewards (punishes) more positive (negative) density inside modules. In this work, we developed specialized algorithms for discovering community structure in signed networks. We first reformulated the Map Equation to measure the quality of partitions, known as Minimum Description Length (MDL). Next, we extended Constant Pots Model (CPM) to collect a spectrum of partitions from highly simplified to detailed ones, by sliding its parameter *λ* from zero to one (Fig. 1a,b). Based on these extensions, the community detection is carried out by minimizing MDL on *λ*-spectrum (Fig. 1c). Moreover, by comparing MDL and the ratio of internal negative (positive) links, the role and topological placement of negative ties can be quantified (Fig. 1d). As the experiments will show, the proposed method is highly reliable on both signed and unsigned networks, overcoming the resolution-limit and inconsistency of Modularity on signed networks. To evaluate the method, we proposed a novel benchmark in which negative ties can be informatively introduced. As a motivation, evaluating the effectiveness of signed detectors is possible only if the negative ties are informative and, to the best of our knowledge, this issue has not been addressed in previous works.

## Results

### Notation

Throughout the paper, the expressions “link,” “edge,” “tie,” and “relation” are used interchangeably, unless we explicitly make a note. A graph *G* is determined by the triple (*V*, *E*, *W*) where *V* is the set of nodes, *E* is the set of edges defined by pair (*v*_*i*_, *v*_*j*_) of nodes ((*v*_*i*_, *v*_*j*_) = (*v*_*j*_, *v*_*i*_) for undirected graphs), and *W* assigns a weight to each edge. The induced graph of node set *a* is defined by (*V*′, *E*′, *W*′) where *i*) *V*′ = *a*, *ii*) (*v*_*i*_, *v*_*j*_) ∈ *E*′, if *v*_*i*_, *v*_*j*_ ∈ *a* and (*v*_*i*_, *v*_*j*_) ∈ *E*, and *iii*) *W*′(*v*_*i*_, *v*_*j*_) = *W*(*v*_*i*_, *v*_*j*_). A partition *C* assigns each node to a module (i.e., *c*_*i*_ is the module of node *i*). Other basic notations are listed in Table 1.

### Tools to explore the community structure of signed networks

In this work, we reformulated the well-known Map Equation *L(G*, *C)* for signed networks, which is the minimum expected code length that is required to address each step of a random-walker (also known as Minimum Description Length). The idea is that negative ties should be used in line with the “random walkers are more likely to be trapped inside a community” intuition. Therefore, a negative tie from node *i* of module *c*_*i*_ towards *c*′ should decrease the probability of going from *c*_*i*_ to *c*′, and conversely, a negative tie towards the inside of *c*_*i*_ should increase the probability of escaping from *c*_*i*_. Accordingly, Map Equation was reformulated to account for the negative ties (see Methods). By increasing the amount of external (internal) negative ties, it is expected that the value of MDL decreases (increases). Also, we extended the Constant Potts Model (CPM) to signed networks. CPM explicitly states that the absence of internal positive ties should be punished using a constant parameter *λ*. In particular, by sliding *λ* from 0 to 1 (or further), the minimization of CPM results in the extraction of smaller and denser modules^16^. Utilizing these well-established foundations, our method provided a map spectrum for signed networks, which not only revealed the best partition of a network but also provided useful information about negative ties on different scales of a network.

### Proposed benchmarks for signed networks

**Figure 2:**
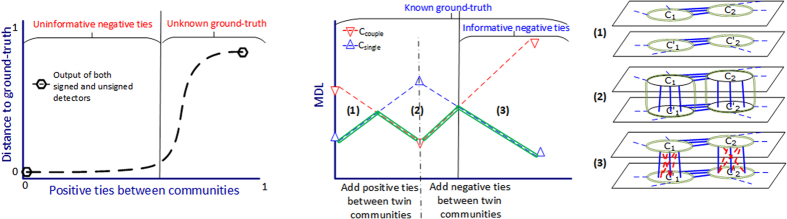
Characteristics of the proposed benchmarks. *C*_*single*_ (*C*_*couple*_) partition places each community of ground-truth (and its duplicate) in one module. (**a**) in signed LFR, for any arbitrary amount of external negative ties, by increasing positive ties between communities, the ground-truth is detectable by unsigned methods, as well as signed ones, until it fades out. (**b**) in coupled LFR, the duplicate (twin) communities are first intertwined with positive ties (phase 1 and 2) and then get separated by negative ties (phase 2 and 3). This leads to (A) a constantly known ground-truth (green double-line) which switches from *C*_*single*_ to *C*_*couple*_ and again back to *C*_*single*_, and (B) informative negative ties in phase 3 where unsigned methods wrongly prefer *C*_*couple*_ to *C*_*single*_. The transition points are illustrated using MDL measure (lower value corresponds to better partition).

In this manuscript, we extended the LFR (Lancichinetti-Fortunato-Radicchi^17^) benchmark to signed graphs. As the main characteristic, our extensions (the same as original LFR) provide scale-free distributions for positive (negative) degrees and community sizes. These benchmarks are denoted as signed and coupled LFR. The signed LFR simply replaces internal (or external) positive ties with negative ones. This extension was used to evaluate the effect of external (internal) negative ties on the extended Map Equation. However, in order to evaluate the power of signed detectors, a network must have two features: (1) a valid ground-truth for comparison, and (2) informative negative ties, which ignoring them leads to incorrect partitioning. These features do not simultaneously hold for the signed LFR, and thus, the coupled LFR was proposed. To show the failure of the signed LFR, the evaluation was started with uninformative negative ties, which can be ignored. Gradually, by placing positive ties between the communities, it was expected that at some point the negative ties become decisive, which means the unsigned detectors unlike signed ones must fail to detect the correct partition. However, before the total collapse of the ground-truth, this did not happen, signifying that the signed LFR has either uninformative negative ties or invalid ground-truth (Fig. 2a). Accordingly, the coupled LFR was introduced. Without going into details, it is composed of two identical LFR graphs, and the merging process occurs only between twin (duplicate) communities (Fig. 2b). As the schematic representation shows, the coupled LFR provides an interval (phase 3) with both informative negative ties and known ground-truth, suitable for evaluating the efficiency of signed detectors. The procedure of creating the benchmarks is as follows:

### Signed LFR

LFR benchmark was extended by introducing external or internal negative ties regarding each node. For the case of external ones, there is an additional parameter 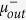, where 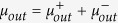, that forces each node to have 
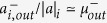
. For example, in a graph with *μ*_*out*_ = 0.6 and 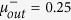, regarding each node *i*, approximately 40% of links are positive and inside module *c*_*i*_, 25% are negative and outside *c*_*i*_, and 35% are positive and outside *c*_*i*_. For the case of internal negatives, using parameter 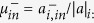, the procedure is the same as the external ones.

### Coupled LFR

This benchmark was built using two identical unsigned LFR graphs that were intertwined using parameters *μ*_*c*_ and 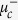, where 0 ≤ *μ*_*c*_ < 1 and 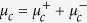. Thus, there are two layers of graphs where each node or community has a duplicate (or twin) in the other one. Keeping the layers unchanged, the benchmark forces node *i* to have 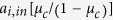 new links towards twin community *c*_*’i*_ (similar for 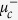). That is, by increasing *μ*_*c*_, twin communities become intertwined, and conversely, by increasing 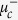 they become separated. However, the nominal *μ*_*c*_ may not be satisfied for some nodes, and thus, the empirical average was reported in the plots. It is worth mentioning that the “twin” notion is not a model of real-world networks, but an easy way of controlling the network structure to produce informative ties (Fig. 2b). In other words, by introducing the coupled LFR, we tried to ensure the effectiveness when negative ties are playing a decisive role in partitioning the network as well as to include some basic characteristics of real-world networks such as scale-free community sizes and node degrees.

### Comparison of partitions

The distance between two partitions *C* and *C*′ was measured using Normalized Variation of Information (NVI). NVI is zero if the partitions are identical, and one if they are statistically independent, meaning no information is gained about *C* by knowing *C*′ and vice versa; see the formulation in Methods.

The notations used for denoting general types of partitions are as follows:

- *C*_*truth*_: ground-truth partition of a graph for [un]signed LFR.
- *C_A_*_*llin*1_: all-in-one partition, which places all the nodes in one module.
- *C*_*single*_: places each community of coupled LFR in one module.
- *C*_*couple*_: places each community and its twin in one module.

### Evaluation of SiMap

**Figure 3:**
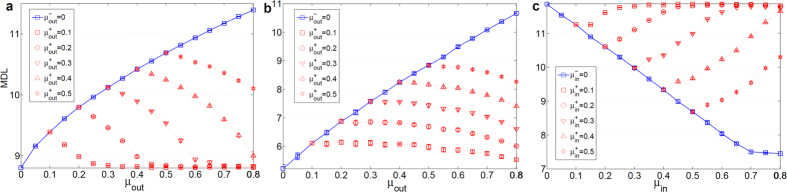
SiMap on signed LFR. (**a**) Network of two communities of size 500. We added the external positive ties until 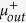, and the negative ties afterwards. Networks with 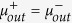 had almost the same quality as the case with 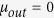. (**b**) Network of many communities with *N*_*G*_ = 1000; although 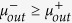, the external negative ties may not cancel the positive flow from each node towards *every* community; therefore, dashed MDL curves dropped slowly, and never reached the level of 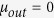. (**c**) Network of many communities with *N*_*G*_ = 1000; the internal negative ties canceled positive ones almost at the same level as 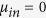. Each value was averaged over 25 graph realizations.

In order to investigate the effect of negative ties, SiMap was examined on signed LFR. In Fig. 3a,b, the internal structure of the communities was kept constant during the increase of mixing *μ*_*out*_, and similarly, the external links were kept constant during the increase of internal ties (*μ*_*in*_) in Fig. 3c. As shown in Fig. 3a, for a network of two communities, when the mixing of only positive ties was increased, the value of MDL (solid curve) increased accordingly, which corresponds to the decrease of quality of communities. Next, we stopped adding positive ties at 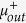 and started adding negative ones afterwards, where 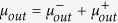. The external negative ties are expected to cancel out the positive ones, and thus the quality of communities increases again (equally MDL decreases) almost to that of *μ*_*out*_ = 0. This expectation was validated using different starting points (dashed curves in Fig. 3a). On the other hand, for a network of more than two communities, randomly-added external negative ties may not cancel the positive ties from each node towards every community. In other words, even if 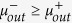, a node may have more positive ties toward a module than negative ones. Hence, as depicted in Fig. 3b, MDL dropped towards the level of *μ*_*out*_ = 0 with a slower slope and never reached that level. This is consistent with our formulation, stating that the external negative ties would cancel all the positive ones, if their weight towards *every* community is, at least, as much as positive ones, otherwise, MDL should be higher than that of *μ*_*out*_ = 0. For the case of internal negative ties, a similar experiment was carried out. As shown in Fig. 3c, internal negative ties canceled the effect of positive ones, and consequently weakened the quality of communities almost to the situation where there had been no ties inside the communities (*μ*_*in*_ = 0).

In general, SiMap not only punishes the presence of internal negative ties, but also rewards the external negative ones. Note that the rewarding is module-wise, which means the mesoscopic topology of the network determines the amount of the reward; e.g., if two modules have no positive ties in between, the inter-negative ones add no information, and therefore have no effect on MDL.

### Map spectrum of CPM(*λ*)

**Figure 4:**
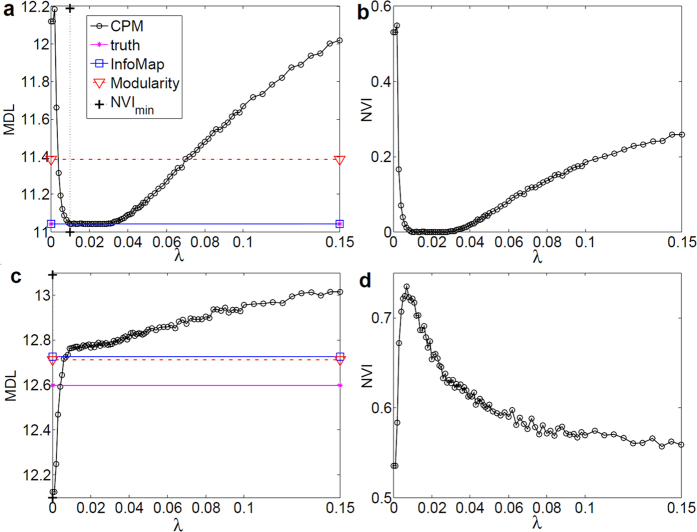
MDL spectrum of CPM(*λ*) on LFR (*N*_*G*_ = 5000). (**a**,**b**) MDL and NVI for 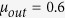; although 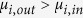 for every node, the community structure is still clear and detectable^24^. (**c**,**d**) MDL and NVI for 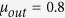, where the network has no significant community structure, and thus single-module has lower MDL than the case when the network is divided into sub-modules.

In this experiment, the SiMap of signed CPM(*λ*) was plotted to illustrate its well-behaved curve with respect to the distance function NVI(*C*_*CPM*_, *C*_*truth*_). For comparison, the statistics of InfoMap (which ignores negative ties) and Modularity were also plotted. As depicted in Fig. 4a, although NVI is aware of the ground-truth partition, MDL curve behaved similarly to NVI, which first smoothly decreased, and then slowly increased. Additionally, the minimum of MDL(*λ*) coincided with the true community structure of the graph. Furthermore, InfoMap reached the same minimum level of MDL, meaning that the negative ties were not informative in this network. On the other hand, at *μ*_*out*_ = 0.8, MDL(*λ*) constantly increased from *λ* = 0 to 1 (Fig. 4c). This indicates that the single-module is preferred to dividing the network into sub-modules, since it has no significant agglomeration of density at this mixing rate^16^.

### Performance of CPMap on Signed LFR

**Figure 5:**
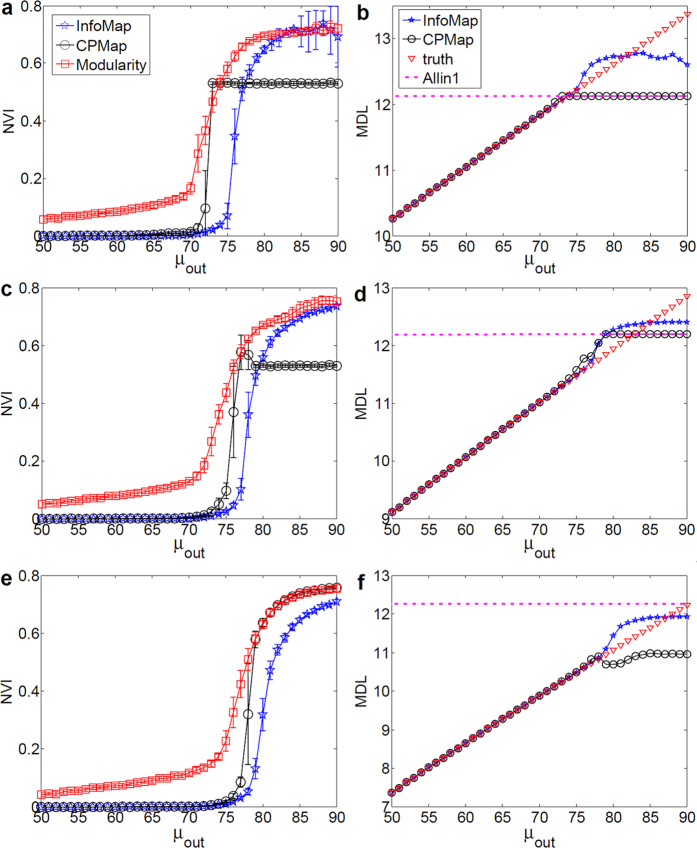
Evaluation of methods on signed LFR (*μ*_*out*_ is plotted in percentage). (**a**,**b**) NVI and MDL of the algorithms for unsigned LFR, (**c**,**d**) NVI and MDL for 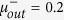, and (**e**,**f**) for 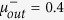. In all cases, CPMap and InfoMap had close performance and both outperformed Modularity. Before 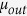 ≃ 0.75, all detectors similarly performed on signed LFR as compared to the unsigned one. Roughly for 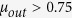, the community structure faded out and all detectors suddenly failed. Therefore, the signed LFR has either non-informative negative ties or invalid *C*_*truth*_. Each value was averaged over 25 graph realizations with *N*_*G*_ = 5000.

In this experiment, we compared InfoMap (which ignores the negative ties), CPMap, and Modularity on signed LFR for 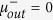 (unsigned), 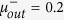 and 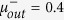. In all cases, CPMap performed better than the signed Modularity. As demonstrated in Fig. 5a,b, on unsigned LFR, CPMap performed nearly as well as InfoMap in optimizing equation (15). This suggests that CPM provides a reach set of partitions. For *μ*_*out*_ ≥ 0.75, CPMap opted for the single-module partition *C*_*Allin*1_, which had a lower MDL than both *C*_*truth*_ and the output of InfoMap. Nevertheless, according to Fig. 5a,d, there still remains a room for future improvements upon CPM to optimize SiMap. In Fig. 5c,d, by adding 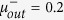 negative ties to each node, even InfoMap was still capable of detecting *C*_*truth*_ before 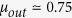. After this threshold, all detectors failed to detect *C*_*truth*_, since the community structure was not valid anymore due to severe rewirings^16^,^18^. In Fig. 5e,f, by adding more negative ties (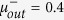), although CPMap reached a better MDL than all other partitions, the situation remained almost the same, that is signed LFR either has non-informative negative ties or invalid *C*_*truth*_.

As a summary, although this benchmark may be first to come to mind, we showed it is not capable of appropriately challenging the signed detectors. The non-informativeness comes from the fact that flipping the external positive links to negative makes the community structure more clear, and thus InfoMap performs on signed LFR as accurate as the unsigned one. For this reason, the coupled LFR was introduced that gives us more leverage on the informativeness of negative ties while keeping the community structure valid.

### Performance of CPMap on Coupled LFR

**Figure 6:**
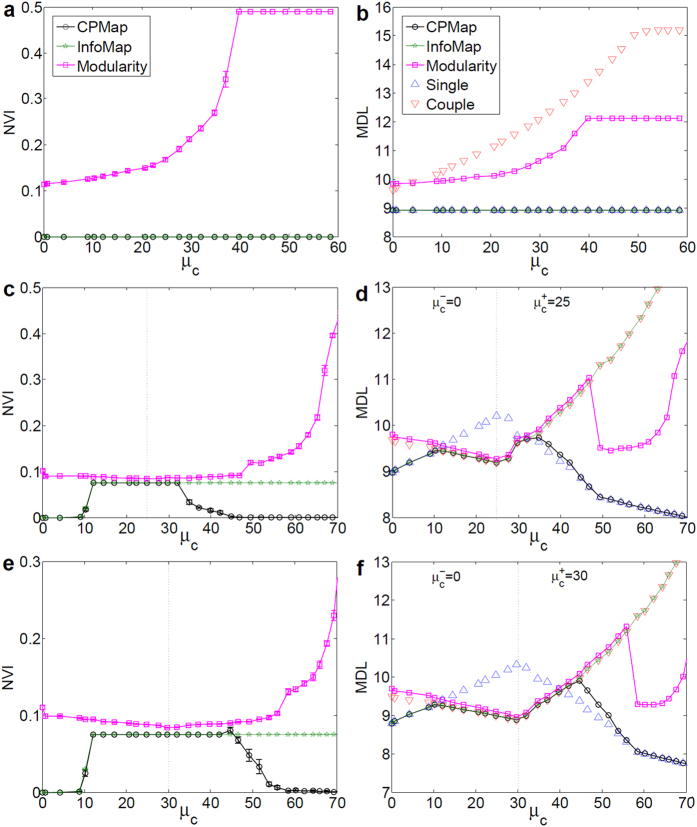
Evaluation of methods on coupled LFR (*μ*_*c*_ is plotted in percentage). (**a**,**b**) Only negative ties were added between two layers. *C*_*single*_ constantly had the lowest MDL and was detected by InfoMap and CPMap. However, the Modularity failed to detect *C*_*single*_ by merging its communities. (**c**,**d**) Positive ties were added between twin communities until 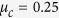, and negative ties were added afterwards. CPMap constantly preferred the structure with the lowest MDL that is, in order, *C*_*single*_, *C*_*couple*_, and again *C*_*single*_. However, InfoMap wrongly preferred *C*_*couple*_ after 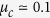, signifying the usefulness of negative ties. (**e**,**f**) The experiment was repeated using more intertwined layers at 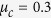. For all cases, Modularity reacted slowly to informative ties, and eventually failed to detect *C*_*single*_. Each value was averaged over 25 graph realizations with *N*_*each layer*_ = 5000.

In this experiment, using coupled LFR, we investigated the ability of CPMap on utilizing the negative ties’ information. To this end, *μ*_*out*_ =0.3 was used for each layer to have connected yet well-separated communities, and only the connectivity of twin communities was manipulated. First, in Fig. 6a,b, two identical layers of graphs were gradually coupled only with negative ties (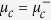). As expected, the output of CPMap and InfoMap constantly resembled *C*_*single*_, since the negative ties added no competing information to the community structure of positive subgraph. However, the output of Modularity, surprisingly, changed with the increase of negative ties. In particular, by increasing *μ*_*c*_, previously detected modules were expanded to form larger ones. In other words, the number of negative ties indirectly weakened the sensitivity of Modularity to the density of positive ties, leading to larger and sparser modules.

Second, in Fig. 6c–f, the amount of positive ties 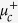 between twin communities was increased until the quality of *C*_*couple*_ surpassed that of *C*_*single*_. At this point, *C*_*couple*_ was preferred by the detectors instead of *C*_*single*_. Knowing that CPMap and Modularity are partitioning the networks based on a criteria other than SiMap, this somehow validated the alignment of SiMap with the true quality of partitions. In the next phase, the negative ties 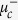 were added between twin communities to break them apart. As a result, the negative ties gradually became informative, since by ignoring them, InfoMap kept partitioning the graph exactly the same as *C*_*couple*_. When *C*_*single*_ surpassed *C*_*couple*_, CPMap started to switch from *C*_*couple*_ to *C*_*single*_ accordingly. However, this switching occurred much later for the signed Modularity, meaning that it is less sensitive to the informative negative ties. Also, it never opted for *C*_*single*_ due to an inherent inconsistency. Nevertheless, there is still a room for improvement upon signed CPM for optimizing SiMap, which is evident from 0.3 < *μ*_*c*_ < 0.45 in Fig. 6c,d and 0.45 < *μ*_*c*_ < 0.55 in Fig. 6e,f, where *MDL*_*single*_ is better than *MDL*_*CPM*_.

### Online Social Media Networks

Using the proposed tools, we investigated the mesoscopic structure of three well-known real signed networks: Slashdot, Epinions, and WikiElections. To this end, we optimized equation (17) for the whole spectrum of *λ* = [0, 1], and further analyzed the corresponding partitions at each scale (only the informative intervals are plotted). In particular, the main information comes from comparing the spectrum of signed CPM to that of CPM+, which is only applied on the positive subgraph, to find the role of negative ties in different scales of the network. Also, this spectrum of partitions were compared with the output of InfoMap and signed Modularity.

**Figure 7:**
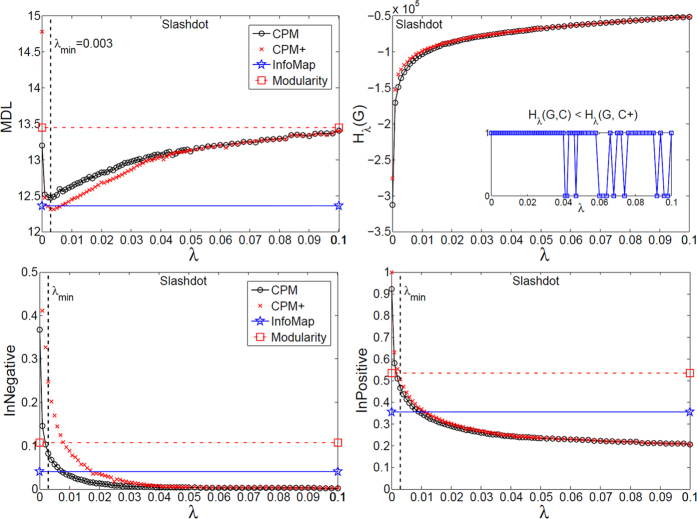
Mesoscopic spectrum of Slashdot dataset. MDL, *H*_*λ*_(*G*, *C*), and the ratio of internal negative (positive) ties are plotted for the output of CPM(*λ*), CPM+(*λ*), InfoMap, and signed Modularity. *λ*_*min*_ marks the best scale for CPM at which it has the lowest MDL. CPM+ is the application of CPM on positive subgraph. If the output of CPM has lower (better) *H* than that of CPM+, the value of inset will be 1, and 0 otherwise.

**Figure 8:**
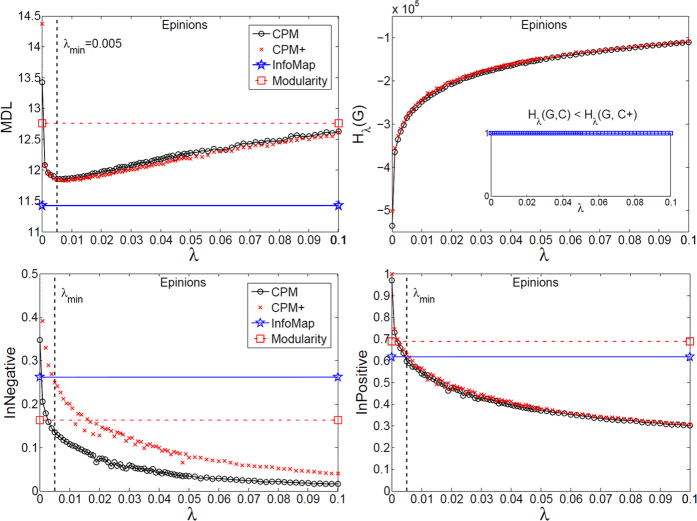
Mesoscopic spectrum of Epinions dataset. Designations are as Fig. 7.

**Figure 9:**
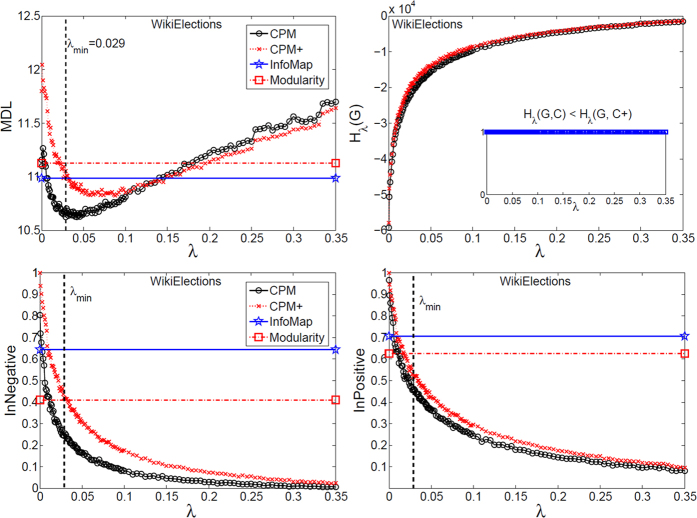
Mesoscopic spectrum of WikiElections dataset. Designations are as Fig. 7.

As depicted in Figs 7, 8 and 9, MDL curve was V-shaped for all three networks with a minimum at *λ*_*min*_, which signifies that *i*) the networks have community structure, and *ii*) the best map of each network is made up of modules with density ≃*λ*_*min*_ or higher that are mutually connected with the same density or lower. In particular, WikiElections had considerably denser modules than both Slashdot and Epinions, which is consistent with its relatively higher density of positive ties (see Table 2). Also, regarding the internal negative (positive) ties, they continuously declined and were placed between the modules, as the interpretation of *λ* suggests.

### Slashdot

According to Fig. 7, in all scales, the MDL curve of CPM+ was better than that of CPM. Also, MDL of InfoMap was better than MDL(*λ*_*min*_) of CPM. Although the optimized value of equation (17) was slightly better for CPM than CPM+, which implies the constructive role of the negative ties in the optimization process, better MDL of both CPM+ and InfoMap signified that negative ties were not informative to achieve a higher quality mesoscopic structure as compared to the coupled LFR. Also, in terms of the internal negative ties, CPM+ placed them between modules after *λ* ≃ 0.015 without using their information, implying that almost all (95%) of negative ties were naturally placed between modules of density ≃0.015 or higher that were mutually connected with the same density or lower. These findings are consistent with previous ones both on microscopic^19^ and mesoscopic levels^20^. However, using the proposed tools, one captures a more quantitative picture of negative ties for the entire spectrum of the mesoscopic structure.

### Epinions

According to Fig. 8, similar to Slashdot, CPM+ and InfoMap reached a better MDL than CPM, meaning that one could not find a better partition by taking negative ties into account. However, CPM+ could not exclude 95% of negative ties until *λ* ≃ 0.085. Therefore, the negative ties are placed between the modules of higher density with stronger interconnections compared to Slashdot. This means that the “negative ties lie between dense positive modules” pattern is apparent, yet, less salient than Slashdot.

### WikiElections

According to Fig. 9, unlike Slashdot and Epinions, CPM had considerably better MDL than both CPM+ and InfoMap at the best scale *λ*_*min*_ = 0.0029 and beyond until *λ* ≃ 0.1. This suggests that the information of negative ties is useful for WikiElections, and only vanishes when one zooms into the network to find the modules of density ≃ 0.1 or higher. Accordingly, negative ties lose their informativeness for *λ* ≥ 0.1. However, this threshold is well before the trivial case *λ* ≃ 1, where the objective is merely to find positive cliques. Additionally, CPM+ could not place 95% of negative ties between the modules until *λ* ≃ 0.25, meaning that the position of negative ties between dense positive ones is considerably less notable than Slashdot and Epinions. It is worth mentioning that Leskovec *et al*. also observed this different pattern of relations from local perspective, which have resulted in a weak cross-generalization of link prediction models, and also less accurate models for WikiElections^21^.

Indeed, these observations can be explained using the intuitive *information trade-off* of negative ties between local level (for sign prediction) and mesoscopic level (for community detection). That is to say, the more principled the negative ties between dense positive regions, the more accurate a link type can be predicted given the information of its neighbors, and conversely, the less information they have for the task of community detection^20^.

### More on Signed Modularity

**Figure 10:**
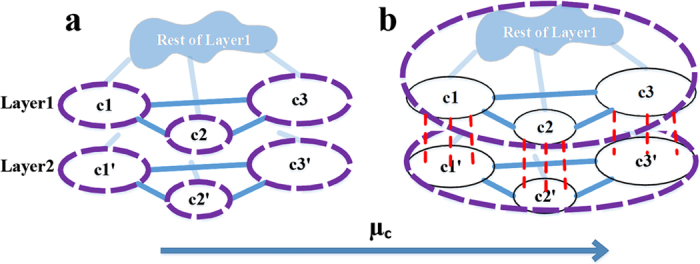
Schematic representation of inconsistency of Modularity on coupled LFR. By adding negative ties between twin communities, eventually, Modularity placed each layer of *C*_*single*_ into one module (**b**).

Based on the results from coupled LFR, by increasing the negative ties, Modularity loses its sensitivity to the density of positive ties. Moreover, our experiments showed that even if two layers of coupled LFR are connected by only negative ties, again, the increase of negative ties leads to placing each layer in one module (Fig. 10b). Note that the coupled LFR is used to resemble two positive regions of a network (with heterogeneous densities internally) that are connected by negative ties. As an attempt to explain this observation, we considered a coupled LFR with fixed parameter *μ*_*out*_, which controls the mixing of each layer, and tunable parameter *μ*_*c*_, which adds [only] negative ties between two layers. Setting 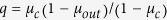, the amount of negative ties relative to positive ones is


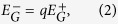


and the sum of positive/negative ties from each module is


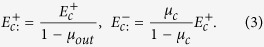


**Figure 11:**
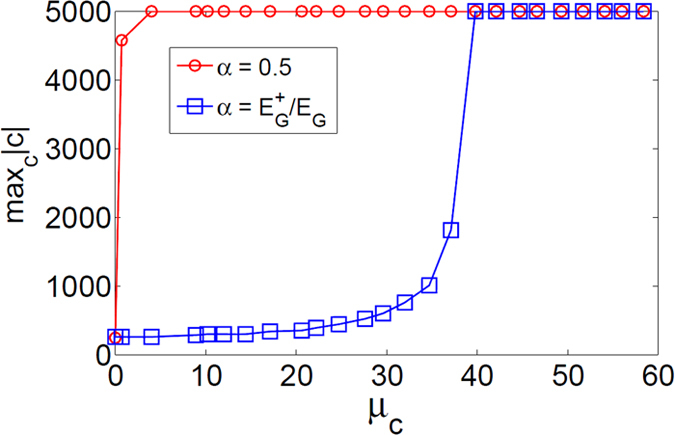
Size of the largest module of Modularity on coupled LFR (*μ*_*c*_ is plotted in percentage). For 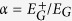, by increasing the number of negative ties between two identical LFR graphs (which have a clear community structure at 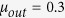), modules were expanded until each layer of 5000 nodes was enclosed in one module. This happened after 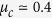 corresponding to 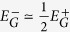. For *α* = 0.5, complete expansion occurred right after a slight deviation from *μ*_*c*_ = 0. The graphs were averaged over 25 realizations with *N*_*each layer*_ = 5000.

Hence, *Q* can be rewritten in module terms as follows:


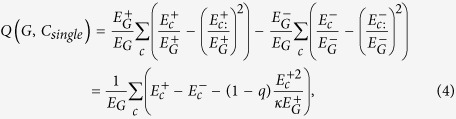


where 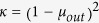 is a constant value. According to the interpretation provided in Methods, the functionality of 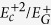, similar to 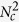, is to control the density of modules. Therefore, by increasing *q*, this sparsity-punishment term is attenuated, and consequently, modules try to gather more links ignoring the loss of density. That is to say, the *density* of each module gradually becomes less important, and conversely, the *number* of internal links becomes more important, which leads to larger and sparser modules consistent with our experiments (Fig. 11). In the same way, equalizing the importance of positive and negative ties (*α* = 0.5) leads to an even worse situation as:


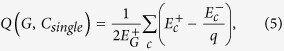


implying that, for any *q* > 0, the objective is to have higher (lower) *number* of positive (negative) ties regardless of the density. This was revealed by our experiments which showed that each layer was placed into one module by a slight increase in *μ*_*c*_ (Fig. 11). In fact, we argue that the reason for this failure is due to the implicit scale of Modularity that is similar for both positive and negative subgraphs. Setting *λ*^−^ = *λ* in equation (16), this becomes more clear as follows:


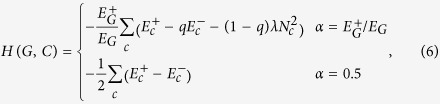


which basically leads the signed CPM to the same drawback as Modularity.

It can be concluded that when the number of negative ties increases, the sensitivity of Modularity to the *density* of positive ties deceases as the objective merely becomes grouping higher *number* of positive ties while excluding the negative ones, which is similar to Correlation Clustering. This inconsistency is resolved for CPM by setting *λ*^−^ = 0 (see Methods).

## Discussion

In this work, we resolved the problem of community detection in networks with positive and negative edges. The proposed algorithm showed an excellent performance on novel synthetic benchmarks. Moreover, it provided a mesoscopic spectrum of signed networks, giving novel insights into the informativeness of negative ties as well as their topological placement between dense positive regions. Hence, one can attain a profound understanding about the structural relevance of positive and negative relations, and utilize that to justify the absorptive-repulsive behavior of the entities according to the context.

The proposed algorithm, CPMap, showed a reasonable performance close to InfoMap on unsigned networks and non-informative signed networks, outperforming signed Modularity. Also, when the negative ties were informative, CPMap performed excellent, extending the capabilities of InfoMap to signed networks. On the contrary, signed Modularity showed considerably weaker sensitivity to the presence of informative negative ties, as well as, growing inconsistency when the relative number of negative ties was increased. This inconsistency was further justified by the physical interpretation of the scale parameter *λ*, shedding new light on the general form of signed objectives.

Regarding the mesoscopic spectrum of real-world networks, we observed that negative ties in Slashdot and Epinions did not contribute to a better quality map than positive subgraph. However, they were informative for extracting the best map of WikiElections, where both CPM+ and InfoMap reached a similar MDL, yet, considerably worse than signed CPM. This usefulness lasted until we zoomed into the network to find the modules of density ≃0.1 or higher. Moreover, the placement of negative ties between dense positive modules was more prominent in Slashdot and Epinions than WikiElections. However, this obscure pattern in WikiElections led to the extraction of more information from negative ties for community detection, consistent with the lower information extracted for the task of sign prediction^21^. Considering the nontrivial position of negative ties, if one wishes to detect modules of maximum density, i.e., positive cliques, negative ties obviously play no role in the detection task, and they are always placed between the modules. However, for Slashdot/Epinions/WikiElections, the majority of the negative ties were between modules of density ≃0.015/0.085/0.25 or higher that were interconnected with the same density or lower, well before this trivial case. Therefore, we showed that it is expected to observe the “negative ties lie between dense positive ties” pattern in a nontrivial setting for real-world networks.

## Methods

### Tools to explore the community structure of signed networks

We first introduce two objective functions used to determine the quality of communities: Map Equation^22^ and Constant Potts Model^23^, which been previously used for unsigned graphs. We reformulated the Map Equation to signed networks (SiMap) by reweighting the walking patterns based on the mesoscopic information of negative ties. Also, we extended CPM to signed graphs, which remains unchanged when the same weight is used for both negative and positive terms. For the final algorithm, the only parameter of signed CPM, *λ*, is determined using SiMap.

### Map Equation

Given a graph *G* and a partition *C*, Map Equation *L(G, C)* is the minimum expected code length that is required to address each step of a random-walker. Suppose a random-walker is going from node *n* to *n′*, this step is addressed as follows:

1. If the walker stays inside module *c*, a code is produced for *n*′.
2. If the walker goes from module *c* to *c*′, an exit-code for *c*, a code for *c*′, and finally a code for *n*′ are produced sequentially.

Accordingly, there are two levels of coding. In the first level a code is assigned to each module, and in the second level each module receives a private coding for members and the action of exiting the module. Finally, using Shannon entropy, the theoretical minimum code length is achieved when the codes are assigned to entities based on their frequency of use. Consequently, the calculation of Map Equation is narrowed down to the relative frequency of visiting nodes and entering-exiting modules. Recent studies have shown that *L* is a very powerful criterion for detection of community structures, both experimentally^24^,^25^,^26^ and theoretically^27^.

### Constant Potts Model (CPM)

To overcome the well-known resolution limit of modularity-alike objectives, Traag *et al*.^23^ suggested an objective function known as Constant Potts Model (CPM) as:


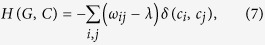


where *λ* is a constant value. This equation can be rewritten in modular terms as:


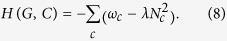


Theoretically, CPM has a clear interpretation based on *λ*^23^; *H* rewards module *c* with density 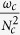 larger than *λ* and punishes *c* otherwise. *H* prefers modules *r* and *s* being separated if they have inter-density 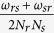 smaller than *λ* and merges them otherwise. We used this interpretation to extend CPM to signed graphs and to analyze real-world networks. Although CPM has a simple formulation, it shows an outstanding performance on the state-of-the-art benchmarks if a proper *λ* is known a priori^23^. However, the burden of community detection is on parameter *λ*. One can get a wide range of partitions from all-in-one to each-in-one by sliding *λ* from 0 to 1 (and even further for weighted graphs). In other words, by increasing *λ*, we zoom into the network to see smaller, denser groups that are interconnected more densely. Consequently, the optimal value of *λ* is a fundamental key to the success of the method.

### Map Equation for signed networks (SiMap)

According to the proposed idea, the information of negative ties should affect the flowing pattern of a random-walker. As a result, given a graph *G* and a partition *C*, the weight (selection probability) of positive ties from node *i* of module *c*_*i*_ towards module *c*′ is first decreased proportional to the negative ties from *i* towards c′, and then the remaining probability (*p*_*i*, *back*_) is channeled back to the internal links. Hence, if a random-walker arrives at node *i*, it is less likely to select the links toward module *c*′ and more likely to step back inside *c*_*i*_. After this, the weight of internal positive ties is decreased proportional to the internal negative ties, and finally, the remaining probability (negative teleport 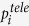), which has been subtracted from the internal positive ties, is uniformly split upon all the nodes in the network. As a summary, in the presence of external negative ties, a walker is less likely to leave *c*_*i*_, and conversely, due to internal negative ties, it is more likely to escape from *c*_*i*_ by choosing a random node outside *c*_*i*_.

Generally, the reweighting process is a heuristic choice. Nonetheless, one can simply make the following assumptions: *i*) if the weight of negative ties toward *c*′ is at least the same as positive ties, the walker should not go to *c*′, and *ii*) if the same situation holds for the links toward the inside of *c*_*i*_, the walker should not directly step back inside *c*_*i*_. Based on these, we propose the following reweighting formulation:


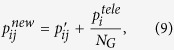


where reweighted (teleport-free) probabilities are


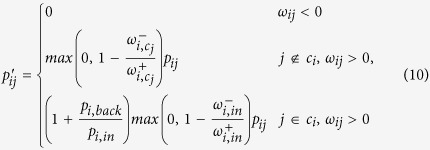


and the negative teleportation from each node is


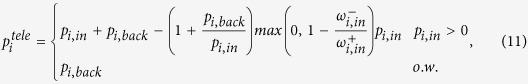


which depends on the backward flow calculated as


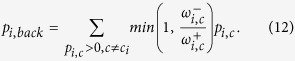


**Figure 12:**
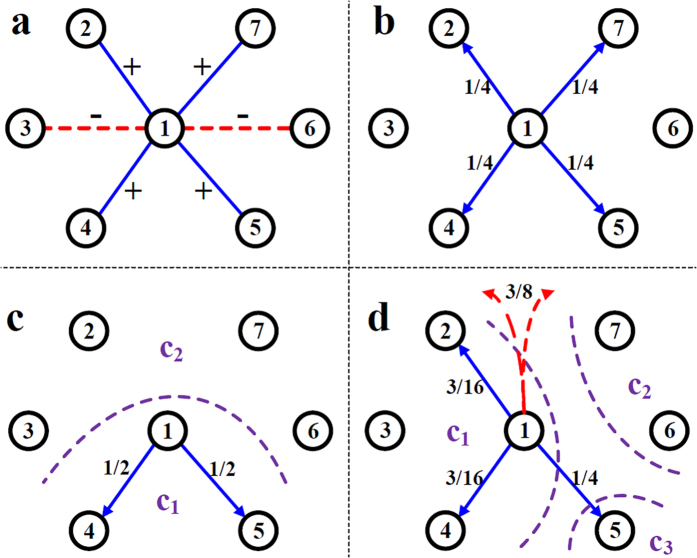
Application of the reweighting procedure on node 1. (**a**) Neighborhood of node 1 in a signed network. (**b**) The flow pattern of positive subgraph from node 1, which is used in the unsigned Map Equation. (**c**) Node 1 has the same amount of negative and positive ties towards *c*_2_, thus, all the flow is channeled back into *c*_1_. (**d**) All the flow towards *c*_2_ is channeled back into *c*_1_, the flow towards *c*_3_ remains unchanged, and 3/8 of flow is uniformly spread over the network due to internal negative tie 1 → 3, which increases both the disorderedness of flow and the probability of escaping from *c*_1_.

To be cleared, two examples of the procedure, which is applied on a sample node, are provided in Fig. 12. As a special case, if there is no negative tie, all transition probabilities remain unchanged.

Now, the probabilities of visiting nodes and entering (or exiting) modules need to be calculated. Note that a graph must be ergodic in order to have a stationary visiting distribution. The ergodicity is guaranteed by the use of teleportation that is being at node *i*, a random-walker either teleports to node *j* with probability *τ *
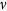
_*j*_ where 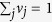, or selects link from node *i* to *j* with probability 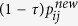
28. Moreover, in an ergodic graph the probability of entering or exiting a module is the same. The probability of visiting node *i* in the reweighted graph *G*′ can be recursively calculated as:


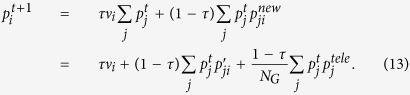


Since the graph is ergodic, starting with an arbitrary distribution, e.g., 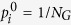, equation (13) converges to the true visiting probabilities [Empirically, distance 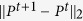 drops to 10^−15^ after around 100 iterations on a graph with 10^6^ links.]. Having *p*_*i*_ calculated for each node *i*, the exiting (or entering) probability of a module *c* is:


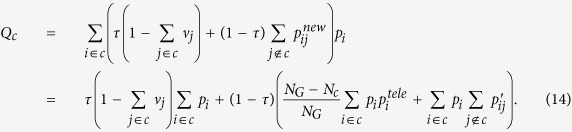


As a special case, if there is no negative tie and the teleportation is uniform 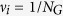, equation (14) is the same as the one derived in^28^.

Having equations (13 and 14) as the main ingredients, the extended Map Equation (SiMap) is calculated as:


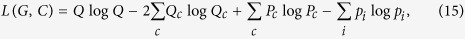


where 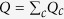 is the probability of using a first-level code, and 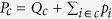 is the probability of using the second-level code of module *c*. Knowing that we encapsulated the information of negative ties in *p*_*i*_ and *Q*_*c*_, equation (15) is the same as that of unsigned graphs^28^.

### Smart teleportation

Lambiotte and Rosvall^29^ showed that although teleportation probability *τ* rectifies the instability of the visiting distribution, it may considerably bias the results. As a solution, they effectively resolved this biased behavior of Map Equation by: 1) setting 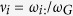, 2) setting *τ* = 0 in equation (14), and 3) iterating equation (13) one extra step using *τ* = 0. In this work, after the reweighting procedure, we did the same for calculation of equations (13 and 14).

### CPM for signed graphs

According to the extension of modularity-alike functions to signed graphs^14^, CPM can be extended as:


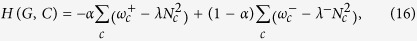


which introduces a new parameter *λ*^−^. In the same work, Traag and Bruggeman manually set non-zero values for parameters *λ* and *λ*^−^ to highlight their importance. Nevertheless, we argue that *λ*^−^ must be set to 0 for the case of CPM. The reason lies in the qualitative objective that is to detect “dense positive” and “negative-free” modules. In particular, “negative-free” condition can be restated as: *any* density of internal negative ties must be punished. However, according to the interpretation of *λ*, if density of the negative ties inside a module *c* is at most *λ*^−^, *c* receives an extra reward from equation (16). This implies that the internal negative ties wrongly increase the quality of *c* compared to the case when they are completely ignored. Therefore, by setting *λ*^−^ =0, *any* amount of internal negative ties is punished. Also, if *α* is set to 0.5 (equal contribution for positive and negative terms), both signed and unsigned CPM objectives will be the same, which only differ in multiplicative constant 0.5:


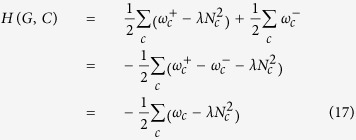


Intuitively, *α* is set to 0.5 to have a certain amount of positive ties being canceled out by the same amount of negative ones. Nonetheless, depending on the application, if intrinsic value of positive ties differ from negative ones, their weights must be set accordingly prior to applying the algorithm.

To optimize CPM, we use an improved version of Louvain method devised by Rosvall and Bergstrom, which is also the one utilized in InfoMap^30^. Louvian method first assigns a unique label to each node, then expands each label to those neighbors that maximally improve the objective value, and finally folds each module into a node and repeats the procedure until no further improvement is made^31^. The improved procedure first runs the Louvain algorithm, and then recursively refines both the nodes and modules to enhance the objective value further^30^. In our experiments, after 3 to 4 refinements the objective value was not considerably improved. Furthermore, the same procedure is used for the Modularity so as to eliminate the potentially biased comparisons due to different optimization procedures.

The main ingredient of Louvain method is the local-update formula^31^. Considering the unsigned CPM, when a set of nodes *κ* is moved from module *c* to *c*′, the local update becomes as^23^:


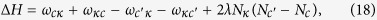


where *κ* is considered in both *c* and *c*′ for calculating *N*_*c*_ and *N*_*c*′_. For the case of signed CPM, the extension is straightforward as:


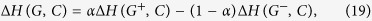


reminding that *λ* is set to zero for the negative subgraph. Regarding equation (19), the positive and negative subgraphs are treated separately during the optimization process.

### Constant Potts Map (CPMap)

SiMap cannot be optimized via local methods of Louvain type, since a local change in a partition costs in the order of total links rather than local links. Indeed, the selection probability of positive ties must be updated according to the new position of negative ties, and thus the stationary distribution needs to be recalculated using equation (13). Nevertheless, SiMap still can be used to select among a set of candidate partitions. In particular, SiMap is used to find the best map of a network among the partitions provided by signed CPM. As Traag *et al*. showed, CPM provides a spectrum of maps that goes from highly simplified to highly detailed by sliding *λ* from 0 to 1^16^. Hence, as the main goal of Map Equation suggests^22^, one can use SiMap to select a map that balances between abridgment and expatiation, while constrained to the “negative free” condition.

Consequently, the proposed algorithm (CPMap) first feeds a set of *λ*s to equation (17), then minimizes the equation to get the corresponding partitions, and finally outputs the one with the lowest SiMap. Candidate *λ*s indeed can be chosen in a number of heuristic ways. However, in the experiments, we had the following observations: *i*) for a network with clear community structure, going from *λ* = 0 to *λ* ≈ 0.1, the MDL curve smoothly dropped, and it slowly rose by further increasing *λ*, and *ii*) for a network with no community structure, the MDL curve rose at the beginning of sliding *λ* away from 0, which means grouping the network as a whole was preferred to dividing it. Based on these observations, the following *λ*-selection is proposed:

1. Set 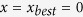 and 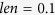.
2. If 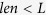, output the partition of 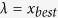.
3. Consider *N* + 1 equally spaced *λ*s in 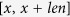. For newly added ones, optimize equation (17) and calculate MDL of corresponding partitions,
  - If *MDL*(*x*) is the minimum, output the partition of *λ* = *x*.
  - Else if 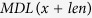 is the minimum, set 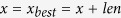, then go to (2).
  - Else if *MDL*(*x*′) is the minimum, set *x*_*best*_ =*x*′, 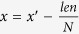 and 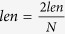, then go to (2).

In the experiments, we set *N* = 4 and *L* = 0.005; since the MDL curve had smooth changes, either increasing *N* or decreasing *L* did not considerably improve the results.

### Normalized Variation of Information (NVI)

This metric^32^ measures the distance between two partitions, which is defined as:


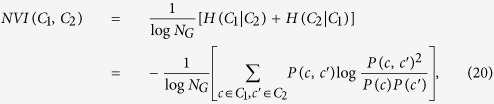


where *H*(.|.) is the conditional entropy, and


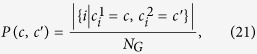


where 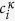 denotes the module of node *i* in partition *κ*. In particular, given one partition, NVI measures the remaining uncertainty about the other one. For example, if *C*_1_ = *C*_2_, given one partition, there is no uncertainty about the other one, and thus *NVI*(*C*_1_, *C*_2_) = 0. NVI is also closely related to Normalized Mutual Information (NMI)^33^; however, NVI is a metric^32^ and has a clear interpretation^34^.

### Common parameters of benchmarks

The parameters for artificial benchmarks were set as: *γ* = 1, *β* = 2, 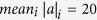, 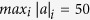, 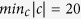, and 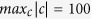. Moreover, regarding the other frequently used settings^24^, the conclusion drawn from each experiment remains the same.

### Online Social Networks and Data preprocessing

We analyze three widely studied online signed social networks: *Slashdot*, *Epinions*, and *WikiElections*^21^. These datasets have been frequently used as benchmarks for studying signed social relations [All datasets are publicly available at http://snap.stanford.edu. For more detailed statistics refer to http://konect.uni-koblenz.de/]. They have special characteristics that make them suitable for the analysis of social relations. For example, all of the links either positive (for friendship or trust) or negative (for enmity or distrust) have been explicitly established by users, which means none of them has been inferred indirectly or asked from a person.

We performed some preprocessings on these datasets making them suitable for our purpose:

1. In order to get an undirected network, reciprocal links with inconsistent signs were omitted, and the remaining links were considered as undirected (inconsistent relations were 0.4%, 0.7%, and 1.6% of relations in Slashdot, Epinions, and WikiElections, respectively).
2. Only the largest connected component of each network was considered (99%, 90%, and 85% of nodes in Slashdot, Epinions, and WikiElections, respectively).
3. Nodes incident to zero positive edges were removed as they, trivially, belong to an isolated cluster.

Table 2 summarizes the properties of these networks after the above operations.

## Additional Information

**How to cite this article**: Esmailian, P. and Jalili, M. Community Detection in Signed Networks: the Role of Negative ties in Different Scales. *Sci. Rep*. **5**, 14339; doi: 10.1038/srep14339 (2015).

## Figures and Tables

**Figure 1:**
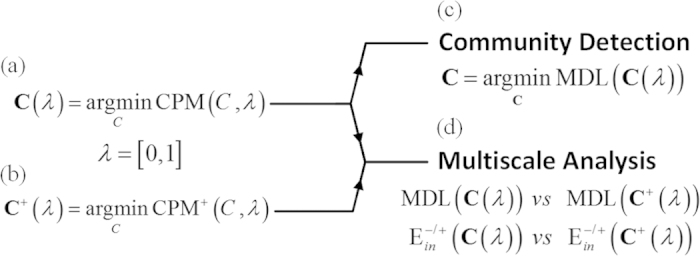
The proposed method. A spectrum of partitions **C**(λ) and **C**^+^(λ) is created by applying the extended CPM on a signed graph (**a**), and its positive subgraph (**b**). The best community is the one with the lowest MDL **(c**). Also, by comparing **C**(λ) and **C**^+^(λ) partitions based on MDL and the ratio of internal negative (positive) links, useful insights about the role and topological placement of negative ties are obtained (**d**).

**Table 1:** Basic notations.

Symbol	Meaning
*E*_*a*_	Number of directed links of induced graph *a*
*N*_*a*_	Number of nodes in *a*
*ω*_*a*_	Sum of directed weights of induced graph *a*
*c*_*i*_	Module of node *i*
*ω*_*ab*_	Sum of weights from *a* to *b*
*ω*_*a*:_	Sum of weights from *a*
*ω*_*i*, *in*_	Sum of weights from node *i* to *c*_*i*_
*ω*_*i*, *out*_	Sum of weights from node *i* to all *c* ≠ *c*_*i*_
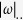	Sum of absolute weights
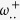	Sum of positive weights
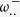	Absolute sum of negative weights
*a*_*ab*_	=sign(*ω*_*ab*_)
*p*_*ab*_	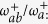
*δ*(*a*, *b*)	=1 if *a* = *b*; =0 otherwise

*a* and *b* could be any set of nodes, but *i* is reserved for a single node. Notations defined for *ω* could be inherited by others.

**Table 2:** Basic statistics of datasets after preprocessing.

	*N*_*G*_	*E*_*G*_/2	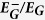	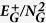
Slashdot	75012	486537	0.2171	1.35E-04
Epinions	103157	668740	0.127	1.10E-04
WikiElections	6521	98907	0.2072	3.70E-02

*E*_*G*_/2 is the number of edges, 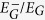 is the ratio of negative edges, and 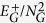 is the density of positive subgraph.
